# Factors associated with poor treatment outcomes among tuberculosis patients in Kyangwali Refugee Settlement, Uganda, 2016–2017

**DOI:** 10.1371/journal.pgph.0000152

**Published:** 2022-08-02

**Authors:** Joyce Nguna, Denis Okethwangu, Steven Ndugwa Kabwama, Dativa Maria Aliddeki, Susan Kizito Kironde, Doreen Birungi, Daniel Eurien, Alex Riolexus Ario, Deus Lukoye, Julius Kasozi, Peter J. Cegielski

**Affiliations:** 1 Uganda Public Health Fellowship Program, Ministry of Health, Kampala, Uganda; 2 Ministry of Health, Kampala, Uganda; 3 United Nations High Commission for Refugees, Kampala, Uganda; 4 Division of Global HIV and TB, Global TB Branch, US Centers for Diseases Control and Prevention, Atlanta, Georgia, United States of America; Universidad Autonoma de Baja California, MEXICO

## Abstract

Communicable diseases, alone or in combination with malnutrition, account for most deaths in complex emergencies including refugee settings. Tuberculosis and HIV/AIDS are increasingly becoming an important cause of morbidity and mortality in refugee settings. We described the treatment outcomes of TB patients and explored factors associated with treatment outcomes among TB patients attending two facilities in Kyangwali Refugee Settlement in Kikuube District, 2016–2017. We abstracted data on laboratory-confirmed patient data from TB registers from 2016 to 2017, in Kikuube Health Centre IV and Rwenyawawa Health Centre II, both located in Kyangwali Refugee Settlement. We abstracted data on socio-demographic variables including age and sex. Other variables were height, weight, final treatment outcomes, demographics, HIV status, TB treatment category, and history of TB. Treatment outcomes were categorized into favorable (including patients who were cured or those who completed treatment) and unfavorable (those in whom treatment failed, those who died, those lost to follow-up, or those not evaluated). We used logistic regression to identify factors associated with unfavorable treatment outcomes. We identified a total of 254 TB patients with a median age of 36 (IQR 26–48) years; 69% (175) were male and 54% (137) were refugees. The median weight was 50.4 kg (range 4–198). Overall, 139 (55%) had favorable outcomes while 115 (45%) had unfavorable outcomes. Refugees formed 53% (71) of those with favorable outcomes and 47% (63) of those with unfavorable outcomes 63(47%). We found that increasing age was statistically associated with unfavorable outcomes, while diagnosis with MDR-TB was associated with decreased odds for unfavorable treatment outcomes. The treatment success rate was lower compared to 85% recommended by WHO. However, the rates are similar to that reported by other studies in Uganda. Innovative approaches to improve treatment success rates with particular focus on persons aged 41–80 years should be devised.

## Introduction

Communicable diseases, account for most deaths in complex emergencies, including refugee settings. Death rates of over 60-fold the baseline have been recorded in refugees and displaced people, with over three-quarters of these deaths caused by communicable diseases [1, 2]. The main causes of morbidity and mortality are diarrheal diseases, acute respiratory infections, measles, and malaria, with HIV/AIDS, and tuberculosis becoming increasingly important [1].

Globally, tuberculosis (TB) is one of the leading causes of morbidity and mortality; despite global efforts geared towards its control, the burden still remains high [3, 4]. Over the past two decades, the number of tuberculosis cases has risen with the increasing population worldwide [5]. In Southeast Asia, incidence peaked around the year 2000 and has been declining slowly, while in Africa, a home to over 11% of the global population had 29% of the global burden of TB and 34% related deaths [5, 6]. The World Health organization (WHO) estimated an average incidence of TB in African countries increased between 1990 and 2005, from 149 to 343 per 100,000 population [7]. However, the incidence of TB has been reducing ever since in all parts of the world.

Like other sub-Saharan African countries, Uganda continues to notify thousands of TB cases although with significant decrease in the number of notified cases [8]. Furthermore, Uganda is one of the few former 22 high-burden TB countries that met all three TB Millennium Development Goal targets, halving the incidence, prevalence, and mortality of TB in 2015 from 1990 levels. According to the national TB indicator survey, Uganda had an annual prevalence of 159/100,000 population in 2015 and 253/100,000 population in 2016 and TB-related mortality at 12/100,000 population [8, 9].

The political unrest in the Democratic Republic of Congo and South Sudan with the resulting violence, economic and social instability, and food insecurity led to an influx of refugees into Uganda from July 2000 until 2017. The United Nation High Commissioner for Refugees (UNHCR) together with the Office of the Prime Minister (OPM) successfully managed to relocate the refugees from Nkondo and Sebigoro landing sites to Kyangwali Refugee Settlement, all in Kikuube District. According to UNHCR, TB accounts for 7% of the causes of death among refugee populations in Uganda [10].

Tuberculosis surveillance is conducted on a weekly basis but according to eHMIS data obtained from DHIS2, these data are not accurate enough to reveal the burden among refugees in Uganda. Furthermore, information about nutritional status and TB among refugee populations in Uganda is limited. High prevalence of under-nutrition in TB patients has been reported in many other countries and Uganda hospital settings and these have been linked to excess deaths and increased risk of relapse [11–13].

There have been few reports documenting the treatment outcomes among TB patients in refugee populations in Uganda. We described the treatment outcomes and identified factors associated with poor treatment outcomes among TB patients in Kyangwali Refugee Settlement, Kikuube, District, 2016–2017.

## Methods

### Study design

We conducted a retrospective cohort study based on patient data from the time of diagnosis of TB to the time of the final outcome. Our study participants were TB patients who attended Kikuube Health Center IV or Rwenyawawa Health Center II during 2016 to 2017.

### Study setting

We conducted the study in Kikuube Health Centre IV, a government-owned facility managing both locals and refugee populations, and Rwenyawawa Health II, which is managed by implementing partners. These are the two health facilities identifying and managing TB patients in Kyangwali refugee settlement, Kikuube District, Western Uganda. Kyangwali refugee settlement, one of the largest settlements in Africa was established in 1960 and currently hosts over 68,703 refugees in a district with 625,568 nationals [10]. The refugees in the settlement account for about 10% of the total district population [10]. The settlement is bordered by Lake Albert in the west with several landing sites; Kabwoya Sub-county to the north; Kigorobya Sub-county to the east; and Kibaale District to the South.

### Study population

We included all records of TB patients registered at Kikuube Health Centre IV or Rwenyawawa Health Centre III during 2016 to 2017, and were recorded in the TB register. To be considered for analysis, patients must have had to have complete demographic information and a treatment outcome.

### Study variables

The dependent variable in our study was TB treatment outcome categorized as favorable (cured and treatment completed) or unfavorable (treatment failed, died, lost to follow-up or not evaluated) and defined by the World Health Organization [14]. The independent variable was weight measurements categorized into more than 50kg and at least 50kgs. Other covariates included HIV-status, smoking status, alcohol use, patient category (new or retreatment), and history of TB.

### Laboratory investigations

Laboratory investigations were conducted in compliance with the national algorithm for TB prevention and control [15].

### Data collection

Using a pretested paper data abstraction tool, we abstracted patient data from TB treatment registers at the two health facilities. Any additional or missing information was obtained from patient treatment charts at the treatment centers.

### Data analysis

We conducted univariate, bivariate and multivariate analysis. In univariate analysis we estimated the distribution of study participants on socio-demographic and other patient characteristics. We determined the median age, and its interquartile range, of participants and percentage distribution for the other characteristics. We presented these in tables and graphs. In the bivariate analysis, we compared outcomes for each predictor variable to determine how risk factors were distributed among the various outcome categories. We conducted multivariable logistic regression to evaluate the independent effects of predictor variables controlling for all covariates reporting adjusted odds ratios (95% CI).

### Ethical considerations

This was a non-research study involving abstraction of routinely collected data. We sought administrative approval to use the records from the Ministry of Health of Uganda (MoH). The Director General of Health Services provided a letter of introduction to the leadership of each facility and permission was obtained from the leadership of all participating health facilities to access patient data. Clearance as a non-research study was obtained from the US Centers for Disease Control and Prevention. Identifying data were removed and all patients were assigned a study ID, to ensure confidentiality. We ensured that all the data were fully anonymized before analysis.

## Results

### Selection of participants

**Fig 1:**
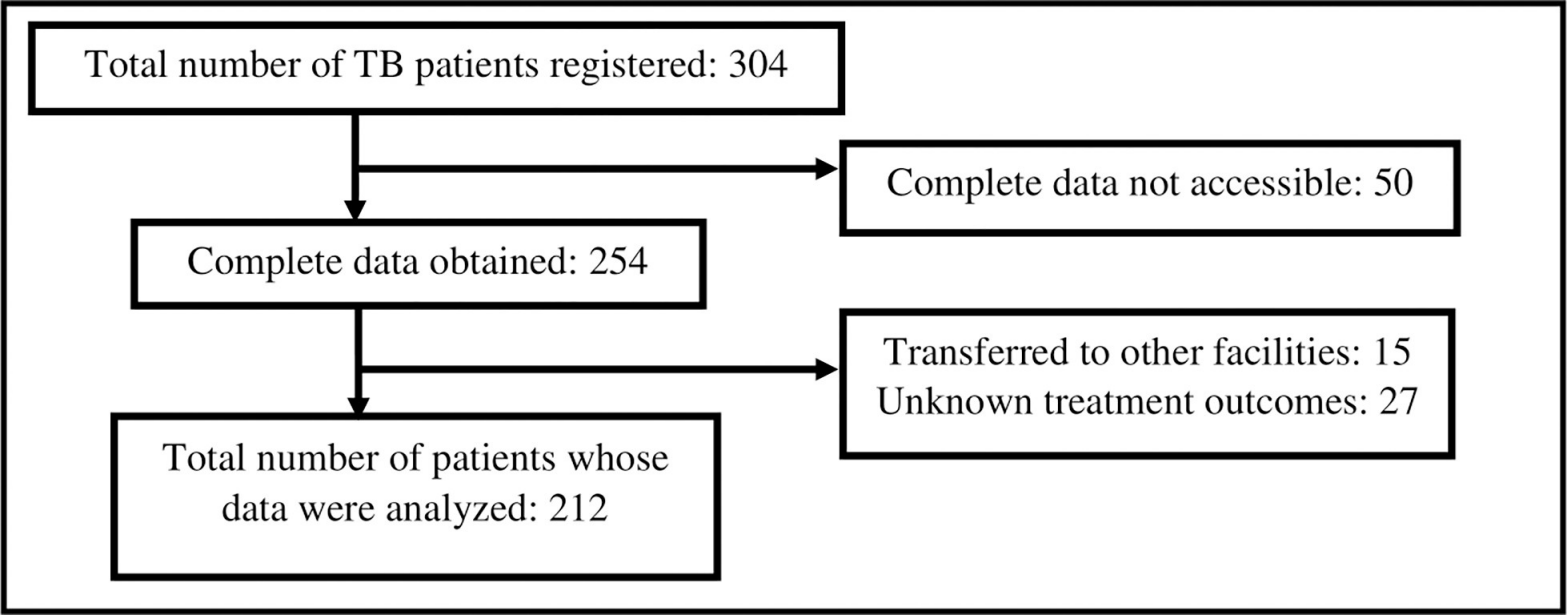
Flow chart of selection of patient records for the study.

During January 2016 through December 2017, we found that 304 TB patients were registered for treatment at the two health facilities in Kyangwali Refugee Settlement. Complete socio-economic data of 50 (16%) of these patients could not be obtained owing to missing information with regard to age, sex, laboratory smear status, type of disease, and treatment outcome. Our analysis also excluded 15 patients who had been transferred to other facilities and 27 others who had unknown treatment outcomes. We therefore conducted analysis on records of 212 (69.7%) patients with complete information **Fig 1**.

### Characteristics of tuberculosis patients managed in Kyangwali Refugee Settlement, 2016–2017

The median age was 36 (IQR: 26–48.5) years; 146 of the 212 participants whose records were analyzed (68.9%) were male, and 109 (51.4%) were refugees. Overall, 41 (19.3%) were HIV positive, and 216 (97%) patients had pulmonary TB; of whom 72 (34.0%) were clinically diagnosed, 134 (63.2%) had been bacteriologically confirmed and 6 (2.8%) had extra-pulmonary TB (EPTB). The largest number of TB patients were in the 21–40 age-group category (94 (44.3%), followed by 41–60 (33.0%), 0–20 (14.6%) and 60–80 (8.0%) with males most affected across all age-groups. There were about the same number of refugees as were nationals (109 refugees vs. 103 nationals) (**Table 1**).

**Table 1:** Characteristics of tuberculosis patients registered for treatment in Kyangwali Refugee Settlement, 2016–2017.

Variable	Characteristics	Frequency	Percent
Sex	Male	146	68.9
	Female	66	31.1
Age group	0–20	31	14.6
	21–40	94	44.3
	41–60	70	33.0
	61–80	17	8.0
Nationality	Native	103	48.6
	Refugee	109	51.4
HIV status	Positive	41	19.3
	Negative	171	80.7
TB history	Pulmonary (Clinical)	72	34.0
	Pulmonary (Confirmed)	134	63.2
	Extra-pulmonary TB	6	2.8

### Treatment outcomes of tuberculosis patients registered for treatment in Kyangwali Refugee Settlement, 2016–2017

Of the 212 TB patients with complete information, 144 (66.5%) completed treatment. None of the patients was categorized as cured. A total of 71 (33.5%) had a poor outcome, including 53 (25%) lost to follow up, 13 (6.1%) died, whereas treatment failed in 5 (2.4%) (Table 2).

**Table 2:** Treatment outcomes among TB patients in Kyangwali Refugee Settlement, 2017–2017.

Categorized outcome	Treatment outcome	n (%)	N (%)
Favorable	Completed treatment	141 (66.5)	141 (66.5)
	Cured	0 (0)	
Unfavorable	Died	13 (6.1)	71 (33.5)
	Loss to follow-up	53 (25.0)	
	Treatment failure	5 (2.4)	

### Factors associated with treatment outcomes among TB patients

At bivariate analysis level, increasing age, i.e. 21–40 years of age (OR = 2.4, 95% CI: 1.12–5.15); 41–60 (cOR = 3.1, 95%CI: 1.38–6.76), and 61–80 (cOR = 5.6, 95%: 1.65–18.9) was the only factor statistically significantly associated with final treatment outcomes. Sex, MDR status, nationality, HIV status, TB history, treatment category, and weight were not statistically significantly associated with treatment outcomes (**Table 3**).

**Table 3:** Characteristics of patients (N = 254) by treatment outcome.

Exposure		Outcome			
		Favorable (n = 141)	Unfavorable (n = 71)	cOR (95% CI)	*p*-value
**Age group**	≤20	13(9.2)	18(25.4)	Ref	
	21–40	61(43.3)	33(46.5)	2.6(1.12–5.87)	0.03
	41–60	53(37.6)	17(23.9)	4.3(1.76–10.6)	<0.01
	>60	14(9.9)	3(4.2)	6.5(1.54–27.18)	0.01
**Sex**	Female	48(34.0)	18(25.4)	Ref	
	Male	93(66.0)	53(74.7)	0.66 (0.36–1.25)	0.35
**Nationality**	Refugee	72(53)	37(47)	ref	
	Native	69(56.7)	34(43.3)	1.04 (0.59–1.85)	0.89
**HIV**	Positive	29(20.6)	12(16.9)	Ref	
	Negative	112(79.4)	59(83.1)	1.27 (0.61–2.068)	0.52
**TB History**	No	109(77.3)	57(80.3)	Ref	
	Yes	32(22.7)	14(19.7)	1.20 (0.59–2.42)	0.62
**Disease Class**	PBC	96(68.1)	38(53.5)	Ref	
	EP*	3(2.1)	3(4.2)	0.40 (0.08–2.05)	0.27
	PCD^#^	42(29.8)	30(42.3)	0.55 (0.30–1.01)	0.05
**MDR status**	MDR	2 (5.9)	3 (2.9)	Ref	
	Non-MDR	32 (94.1)	92 (97.1)	2.06 (0.33–12.90)	0.44
**Patient type**	New	109(77.3)	57(80.3)	Ref	
	Relapse	32(22.7)	14(19.7)	1.19 (0.59–2.42)	0.62
Smear status*	1+	46(32.6)	21(29.6)	Ref	
	2+	41(29.1)	25(35.2)	0.75(0.37–1.53)	0.43
	3+	50(35.5)	17(23.9)	1.66 (0.79–3.51)	0.18
**Weight**	<50Kg	66(46.8)	42(59.2)	Ref	
	>50Kg	75(53.2)	29(40.9)	1.57(1.07–2.31)	0.22

*There were 12 cases with missing smear results.

## Discussion

In Kyangwali Refugee Settlement, our study indicates that there were favorable outcomes among two-thirds of TB patients who sought care during January 2016 to December 2017. Majority of these TB patients had been bacteriologically confirmed though some had been clinically diagnosed and a few more diagnosed with extra-pulmonary TB. We found that with increasing age, the more likely patients were to have unfavorable treatment outcomes to TB. We also found that the odds of having unfavorable treatment outcomes among non-MDR-TB patients were higher compared with MDR-TB patients, though this was not statistically significant.

The proportion of patients with favorable treatment outcomes in the Kyangwali Refugee Settlement was lower than those reported among disadvantaged communities in India; among the homeless in Portugal; patients in Mogadishu, Somalia and several other countries [16–22]. A study in Uganda conducted among 18 sites affiliated with the National Tuberculosis and Leprosy Program of Uganda found a significant variation in treatment outcomes (ranging from 42.6% to 87.6%), with a pooled favorable treatment rate of 69.4%, a figure not very different from that in our study [23]. However, a separate study conducted in Kampala, Uganda reported favorable treatment outcomes in 80.0% of TB patients [24].

Older patients in a large cohort study conducted in Finland were found to be at a significantly higher risk of death from TB [19]. This is supported by a study conducted to evaluate TB programs among the homeless in Portugal and yet another study that reviewed data from the National TB Surveillance System in the United States; both of which found age to be a predictor of unfavorable TB treatment outcomes [20, 21]. Older persons are generally considered vulnerable because of the attendant co-morbidities, which may often be associated with age, and the expected physiological deterioration [25]. A study conducted among homeless TB patients found that the odds of unfavorable treatment outcomes increased by 3% for each 1-year increase in age [21]. With old age, there is a generally increased need for close monitoring of health. However, this may not be easy for older persons considering the challenges of access to healthcare among refugees in settlements, including Kyangwali [26].

Though not statistically significant, the study finding that an MDR-TB diagnosed patient had lower odds of unfavorable treatment outcomes than that among non-MDR-TB patients was surprising. This may have been attributed to the low numbers of MDR-TB patients in our study. A secondary data review in the United States indicated that patients diagnosed with MDR-TB had higher odds for unfavorable outcomes (death) compared with those without [20]. Considering that treatment of MDR-TB is more cumbersome compared to pan-susceptible TB, it would have been expected that unfavorable treatment outcomes would be more significantly associated with MDR-TB [27]. However, management of MDR-TB in Uganda has gained traction and support by both government and partners has been improved. We speculate that the management strategy, which includes admission of MDR-TB patients for the initial phase of their treatment, and close monitoring even after discharge, may have contributed to these findings.

Our study had the following limitation: incompleteness of patient information on key variables, including nutritional status, smoking behavior, marital status etc. made it impossible to evaluate variables known to influence TB treatment outcomes. One strength of the study was that the data collected were over 2 years, which gave the study the power required to validate our findings.

## Conclusion and recommendations

Unfavorable treatment outcomes among patients of TB in Kyangwali Refugee Settlement were more likely to occur among older persons as compared to those in the 20-24-year-old category. The older the patient was, the higher the likelihood for unfavorable treatment outcomes. Patients diagnosed with MDR-TB were less likely to experience unfavorable treatment outcomes as compared to those who were weakly microscopically confirmed. These findings are important for the Ministry of Health and implementing partners working with refugees to the extent that there is need to enhance access to healthcare to all categories of the population. A similar approach taken in the management of MDR-TB should be adopted among TB patients.

## List of abbreviations

ART: Anti-Retroviral Therapy
CI: Confidence Interval
cOR: crude Odds Ratio
aOR: Adjusted Odds Ratio
SD: Standard Deviation
TB: tuberculosis
HIV: human Immunodeficiency Virus
WHO: World Health Organization
